# Characterization of the complete chloroplast genome of mangrove *Rhizophora apiculata* Blume (Rhizophoraceae)

**DOI:** 10.1080/23802359.2021.1923421

**Published:** 2021-06-23

**Authors:** Yi-Chan Li, Su-Yuan Li, Tian-Hao Zhang, Lan-Li Qin, Yi-Dong An, Yu-Kun Pang, Guo-Feng Jiang

**Affiliations:** Guangxi Key Laboratory of Forest Ecology and Conservation and State Key Laboratory for Conservation and Utilization of Subtropical Agro-Bioresources, College of Forestry, Guangxi University, Nanning, PR China

**Keywords:** Mangrove, *Rhizophora*, plastid genome, evolution

## Abstract

The chloroplast (cp) genome sequence of *Rhizophora apiculata* was characterized. The cp genome length was 164,343 bp in length, containing a typical structure of a large single copy (LSC) of 93,155 bp, a small single copy (SSC) of 19,376 bp, and two inverted repeats (IRs) of 25,906 bp, with a GC content of 34.9%. There were 131 genes were annotated in the cp genome, including 85 protein-coding genes, 38 tRNA genes, and 8 rRNA genes. A phylogenetic analysis using cp genomes of mangroves and ecologically associated species resolved *R*. *apiculata* in *Rhizophora* with *R. stylosa* and *R.* x *lamarckii*. This complete chloroplast sequence offers a promising tool for further species identification and evolutionary studies of *Rhizophora*, as well as for mangroves.

## Introduction

Mangroves are a diverse group of woody trees and shrubs that thrive in saline and daily inundation conditions in the inter-tidal zone of tropical and subtropical regions (Ball 1988; Duke 1992). Mangrove ecosystems are among the most carbon-rich forests in the tropics (Donato et al. 2011; Alongi 2014), not only providing the services for lots of animals, but also providing protections from erosion, storm surge, and potentially tsunami waves (Gedan et al. 2011). Mangroves are threatened by climate changed-induced drought, as well as, relative sea level rise (Lovelock 2020; Saintilan et al. 2020). Mangrove forests have been severely degraded over the past half century (Blasco et al. 2001; Duke et al. 2007; Donato et al. 2011), injecting new urgency into understanding the bio-resources of mangroves.

*Rhizophora* consists of five species and at least 6 hybrid taxa, which are largely separated into Old and New World groups (Schwarzbach 2014; Chen et al. 2015). The Old World taxa (*R. apiculata, R. mucronata*, *R. stylosa*, *R.* x *annamalayana*, *R.* x *lamarckii*) are considered the most important of all mangroves across the Pacific tropical region (Duke 2006, 2010). Therefore, providing the genome sequences of species *R. apiculata* will help to spur research on their interesting adaptations, and also could offer some needed information for its conservation. Chloroplast DNA (cpDNA) could provide useful and abundant information on genetic diversity and evolution based on our previous studies (Jiang et al. 2016; Lei et al. 2018), as well as in other mangrove species (Chen et al. 2019; Yang et al. 2019; Shi et al. 2020). In this study, we assembled and characterized the chloroplast genome of *R. apiculata* based on Illumina pair-end data, and built a phylogenetic tree using plastomes available in mangroves and ecologically associated species.

Total genomic DNA was extracted from frozen fresh leaves collected in Sanya Tielu Port mangrove reserve (Sanya, P. R. China, 18° 15′ N/109° 42′ E), following previously described methods (Jiang et al. 2016; Lei et al. 2018). The voucher (*R. apiculata*_Jiang_R9) is stored in Guangxi University, plant ecophysiology and evolution research group herbarium. A 350-bp paired-end library was then constructed and sequenced by Novogene (Beijing, PR China) using an Illumina HiSeqX-ten system (Illumina, San Diego, CA). After sequence filtering for read quality, about 1 Gb raw data were obtained, with a paired-end read length of 2 × 150 bp. A *de novo* assembly was performed using NOVOPlasty3.6 (Dierckxsens et al. 2016), using a seed sequence from the mangrove *Avicennia marina* (AB114520.1). The assembled sequence was then imported into Geneious R9 (Biomatters Ltd, Auckland, New Zealand), to check manually as described previously (Jiang et al. 2016; Hinsinger and Strijk 2017; Xu et al. 2017, 2018). The minimum coverage for the assembled cp genome was 48 ×, and annotations were then transferred from *Rhizophora* x *lamarkii* (NC_046517). The final annotations were confirmed and integrated from results of CPGAVAS2 (Shi et al. 2019) and IRscope (Amiryousefi et al. 2018).

The assembled cp genome of *R. apiculata* had a length of 164,343 bp (GenBank accession number MW387538). The cp genome exhibited a typical structure with a LSC of 93,155, a SSC 19,376 and two inverted repeats (IRa and IRb) of 25,906 bp, respectively. The overall GC content of the plastome was 34.9%, while the GC content in LSC, SSC, IRa, and IRb regions were 32.2%, 28.5%, 42.2%, respectively. In total, 131 genes were annotated, including 85 protein-coding genes, 38 tRNA genes, and 8 ribosomal RNA genes. Seventeen genes (*atpF*, *clpP*, *petB*, *petD*, *rpl16, rps12, rpoC1*, *ycf3*, *trnG-UCC*, *trnK-UUU*, *trnL-UAA*, *trnV-UAC* in LSC; *ndhA* locates in SSC; *ndhB*, *rpl2*, *trnA-UGC*, *trnI-GAU* in the IRs regions) contain 1–2 introns, respectively. While 17 genes were duplicated in the IR regions, including 6 protein-coding genes (*rpl2*, *rpl23*, *ycf1*, *ycf2*, *ndhB*, *rps7*), 7 tRNA genes (*trnA-UGC*, *trnI-GAU*, *trnI-CAU*, *trnL-CAA*, *trnN-GUU*, *trnR-ACG*, *trnV-GAC*), and 4 rRNA genes (4.5S, 5S, 16S, 23S). Notably, sequence of the translation initiation factor gene (*infA*) was found located within *rps8*, with the existence of several internal termination codons indicating a functional loss of *infA* in the cp genome of *R. apiculata*.

**Figure 1.**
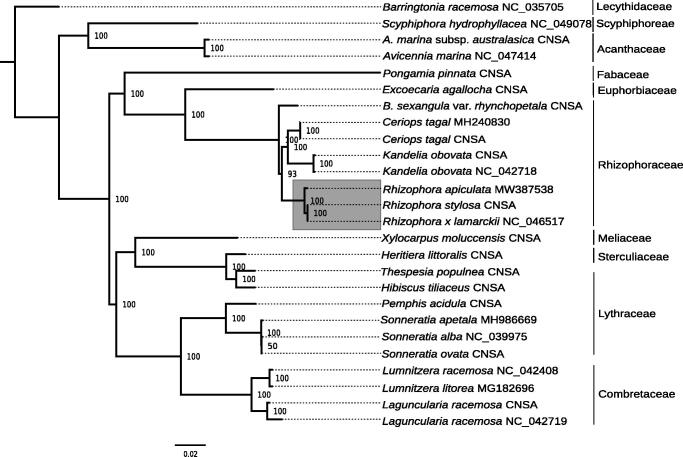
ML phylogeny of mangroves and ecologically associated species based on 25 cp genomes retrieved from the present study, GenBank, and CNSA. The tree is rooted with *Barringtonia racemosa*. Bootstraps values (1000 replicates) are shown at the nodes. Scale in substitution per site.

Twenty-four plastomes of mangroves and ecologically associated species were retrieved from GenBank (accessed 2020/11/17), and CNSA (https://db.cngb.org/cnsa/) according to the reference (Shi et al. 2020), plus *Barringtonia racemosa* as an out-group (Figure 1). All the cp genome sequences were aligned with MAFFT v7.307 (Katoh and Standley 2013), and a maximum likelihood (ML) tree was built using PhyML v3.3 (Guindon et al. 2009) with a GTR + I + G model and support estimated with 1000 bootstraps replicates. All but one node were highly supported (bootstrap support ≥93), and *R. apiculata* clustered with the other two species of *Rhizophora*. The results indicated that providing the plastome of *R. apiculata* will be useful bioresource that will help to assess species identification and further genetic studies in mangroves.

## Data Availability

The genome sequence data that support the findings of this study are openly available in GenBank of NCBI at (https://www.ncbi.nlm.nih.gov/) under the accession no. MW387538. The associated BioProject, SRA, and Bio-Sample numbers are PRJNA713533, SRR13933380, and SAMN18253766, respectively. Data were also available in the database: CNGB Sequence Archive (CNSA) of China National GeneBank DataBase (CNGBdb) with accession number CNP0001525 (https://db.cngb.org/search/project/CNP0001525/).
